# A Rare Presentation of Priapism From Pegylated Filgrastim During Treatment of Urothelial Cancer in an African-American Patient With Sickle Cell Trait

**DOI:** 10.7759/cureus.84492

**Published:** 2025-05-20

**Authors:** Nikhita Kathuria-Prakash, Leland Damron, Sandy C Hernandez, Steven D Hart, Alexandra Drakaki

**Affiliations:** 1 Hematology and Medical Oncology, University of California Los Angeles David Geffen School of Medicine, Los Angeles, USA; 2 Medicine, University of California Los Angeles David Geffen School of Medicine, Los Angeles, USA; 3 Pathology and Laboratory Medicine, University of California Los Angeles David Geffen School of Medicine, Los Angeles, USA

**Keywords:** hemoglobinopathy, pegylated filgrastim, sickle cell trait, stuttering priapism, vaso-occlusive crisis

## Abstract

Priapism is a complication of sickle cell disease resulting in organ-threatening vaso-occlusive crisis and has been reported rarely in patients with sickle cell trait. We report a case of a patient with urothelial carcinoma undergoing chemotherapy and growth factor support with pegylated filgrastim with stuttering priapism triggered by pegylated filgrastim. This is the first case reported, to our knowledge, of stuttering priapism triggered by pegylated filgrastim, and we hope to raise awareness of this potentially organ-threatening complication in this vulnerable population.

## Introduction

Priapism is a manifestation of vaso-occlusive crisis associated with sickle cell disease but has also been reported with sickle cell trait [1,2]. Patients with sickle cell trait are at higher risk for priapism than the general population [1,2], but priapism is typically triggered by trauma, vasodilatory medication, or arousal. The pathophysiology of vaso-occlusive crises such as priapism is similar in both sickle cell disease and sickle cell trait and is caused by the sickling of red blood cells due to a mutation in one or both copies of the gene encoding hemoglobin in sickle cell trait and sickle cell disease, respectively. Given the propensity for patients with sickle cell trait to develop atypical vaso-occlusive crises, it is important for physicians of all subspecialties to be aware of these potential organ-threatening complications. We describe a case of a patient with sickle cell trait who had two episodes of stuttering priapism in the week following pegylated filgrastim growth factor support after adjuvant cisplatin and gemcitabine for urothelial carcinoma and aim to increase awareness of this potential adverse effect in this patient population.

## Case presentation

**Figure 1:**
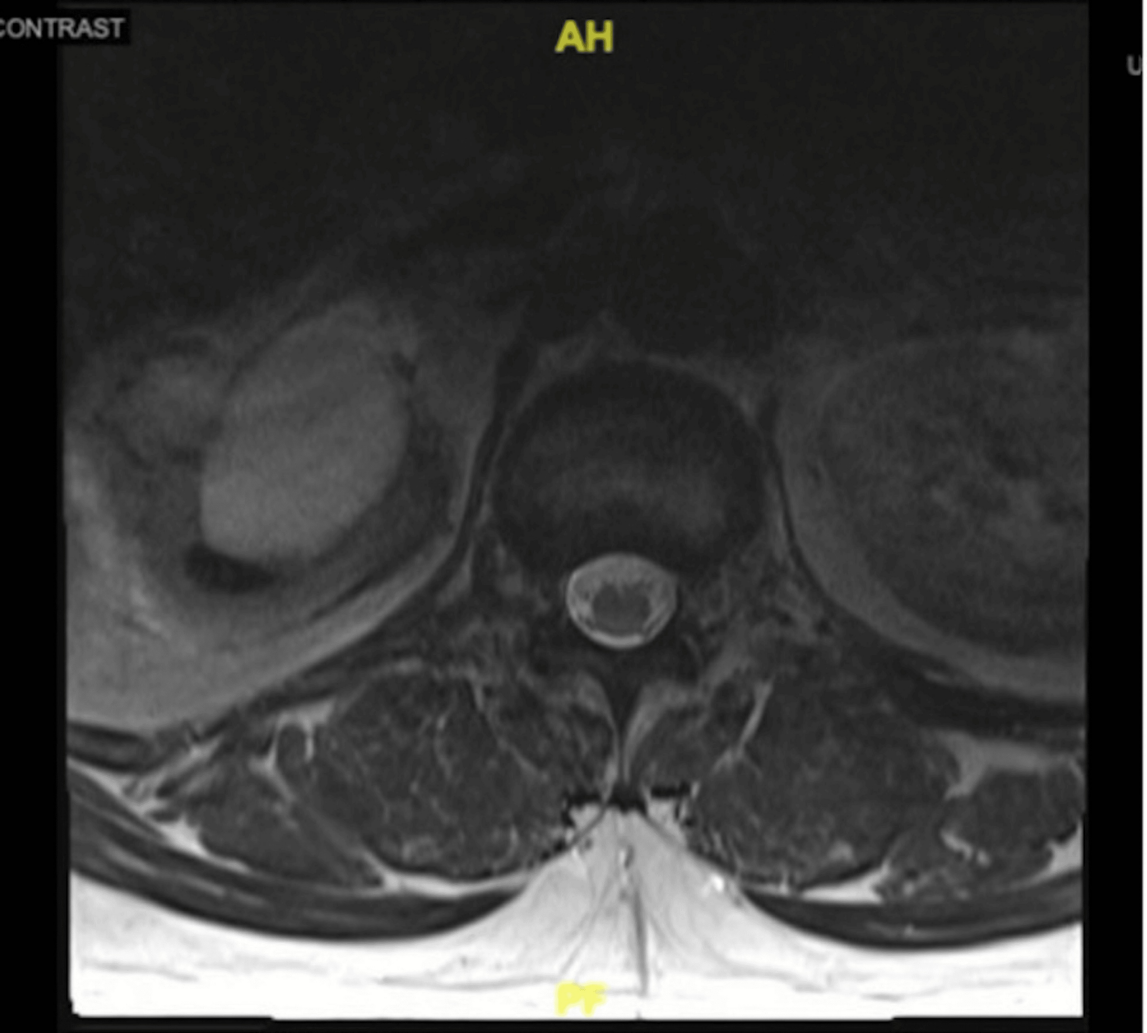
T2-weighted MRI showing an abnormal right kidney with hydronephrosis.

**Figure 2:**
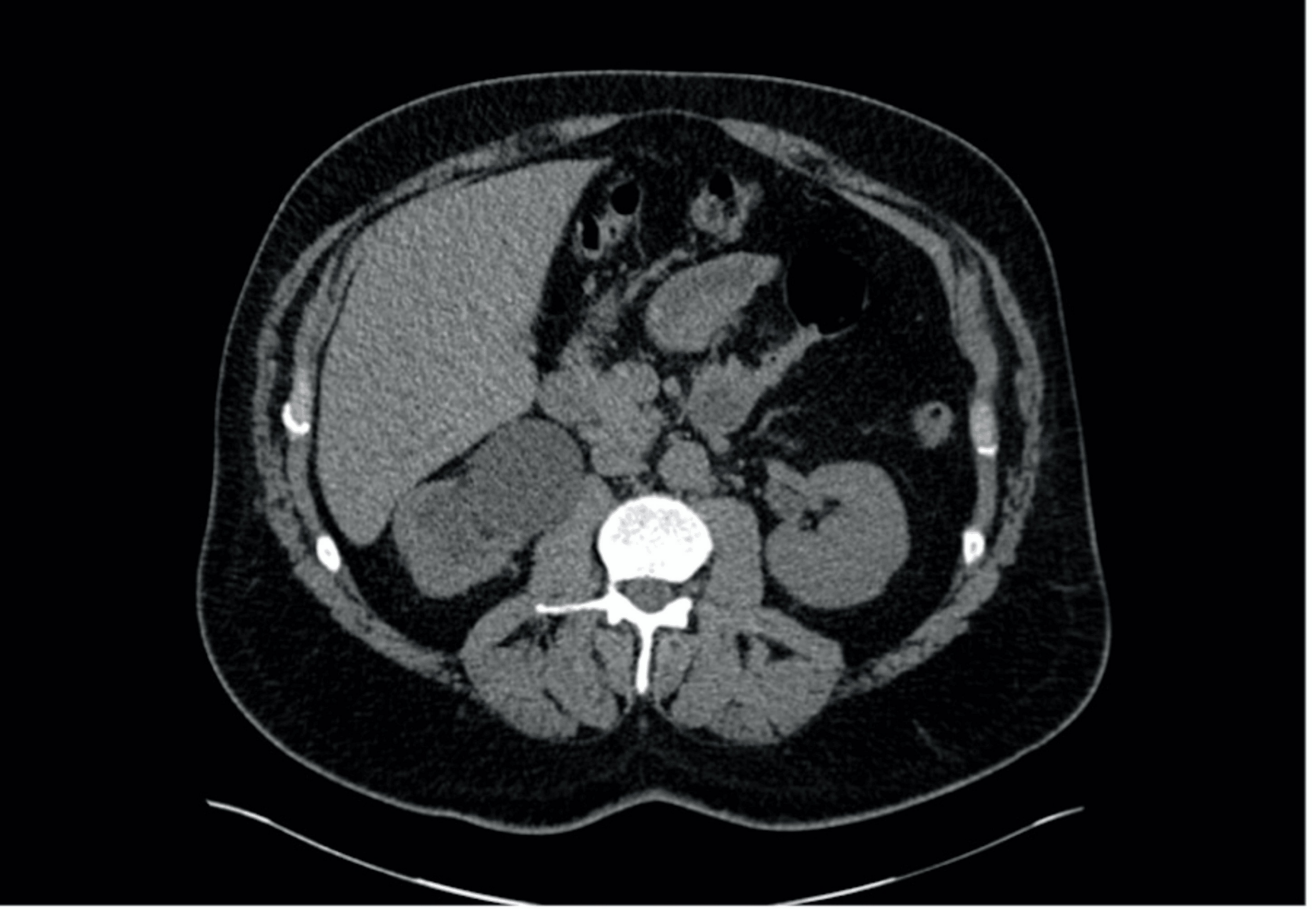
CT kidney ureter bladder (KUB) demonstrating severe chronic right hydronephrosis and proximal hydroureter with long segment thickening and stricture at the mid-distal ureter. Adjacent subcentimeter lymph nodes and periureteral fat stranding with moderate cortical thinning.

**Figure 3:**
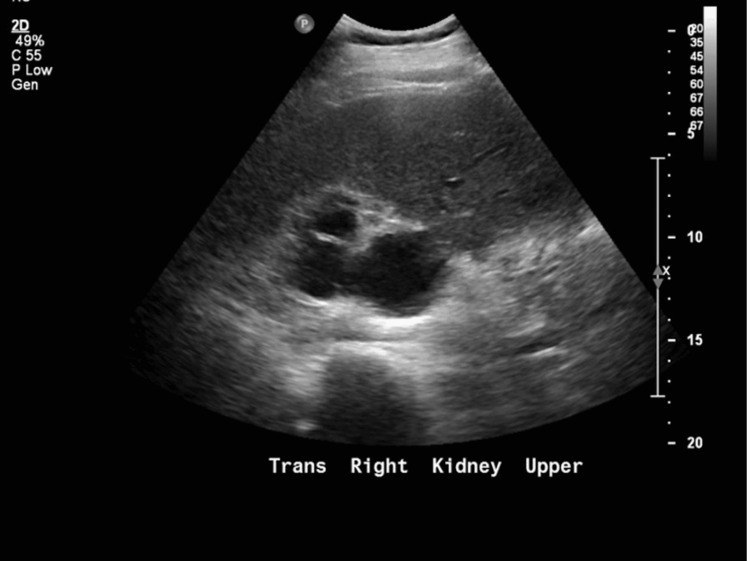
Renal ultrasound showing severe hydronephrosis with moderate cortical thinning and hydroureter.

**Figure 4:**
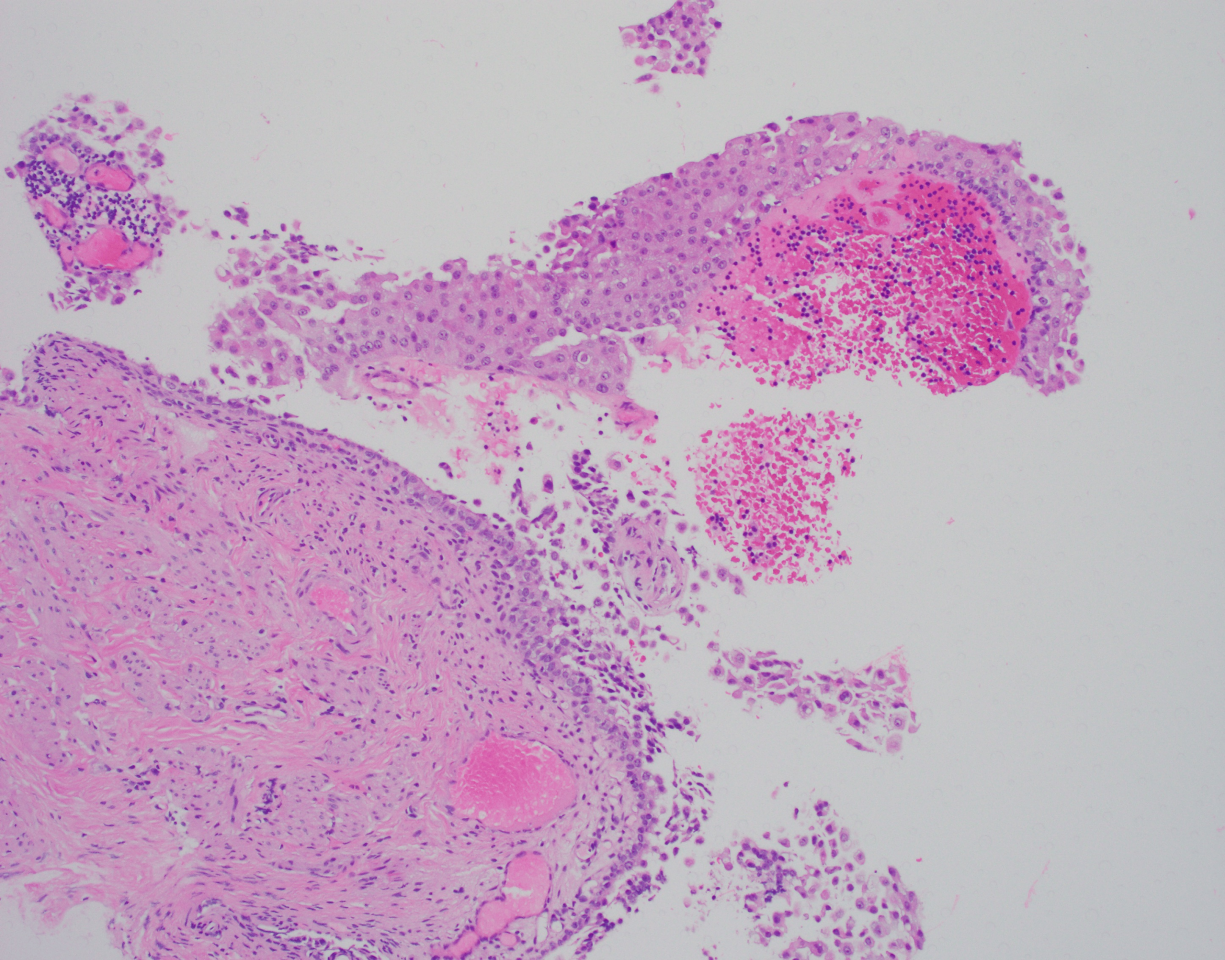
H&E stained slide from bladder biopsy, magnification of 100x, depicting high-grade papillary urothelial carcinoma.

A 64-year-old African American male with a history of multiple sclerosis (MS) on peginterferon beta 1a, stage 3a chronic kidney disease, hypertension, hyperlipidemia, gastroesophageal reflux disease, obstructive sleep apnea, and erectile dysfunction, a potential risk factor for priapism, was diagnosed with invasive high-grade urothelial carcinoma of the right upper urinary tract. An MRI of the thoracic spine for MS surveillance incidentally noted right-sided hydronephrosis, and CT and ultrasound of the kidney confirmed hydronephrosis without a clear etiology of obstruction (Figures 1-3). He underwent cystoscopy and right-sided ureteroscopy and was found to have a ureteral papillary tumor, with pathology demonstrating high-grade papillary urothelial carcinoma. He underwent robot-assisted right nephroureterectomy that revealed a pT3Nx invasive high-grade urothelial carcinoma with focal micropapillary features and negative surgical margins (Figure 4). Given his moderate renal insufficiency, he was recommended four cycles of adjuvant split-dose cisplatin and gemcitabine, with the split dosing to minimize potential nephrotoxicity of cisplatin. He was noted to have microcytic anemia (Table 1) with hemoglobin of 11.7 g/dL and mean corpuscular volume of 74.2 fL, so hemoglobinopathy screen was sent to evaluate for thalassemia. Hemoglobin electrophoresis (Table 1) was consistent with sickle cell trait, with the high Hgb A/S ratio suggestive of concurrent alpha thalassemia minor.

**Table 1:** Complete blood count and hemoglobin electrophoresis results.

Lab	Patient’s Value	Normal Range
Hemoglobin	11.7 g/dL	13.6-17.1 g/dL
Mean Corpuscular Volume	74.2 fL	79.3-98.6 fL
Hemoglobin S	33.9%	0%
Hemoglobin A2	4.5%	2.0-2.9%
Hemoglobin A	61.6%	96-97.9%
Hemoglobin F	0%	0-1.1%

Prior to initiation of chemotherapy, an extensive discussion regarding the risks of chemotherapy and filgrastim administration in a patient with sickle cell trait and MS was conducted with the patient. After discussing the risks and benefits, he began cycle one of standard protocol split dose cisplatin and gemcitabine and received prophylactic pegylated filgrastim. In the week following pegylated filgrastim, he noted several spontaneous non-painful erections each of which lasted three hours and had near emergency care visit. A detailed history was performed and other risk factors and etiologies of priapism, such as phosphodiesterase-5 inhibitors, trauma, and arousal, were not present. Therefore, due to his hesitance over possibly triggering a sickle cell crisis, which he had not experienced before, he opted against prophylactic pegylated filgrastim growth factor support on future cycles. Rather, he opted for close surveillance of his blood counts and administration of filgrastim only if he developed neutropenia. He successfully completed four cycles of chemotherapy without further episodes of priapism, while neutropenia was managed with filgrastim when needed, which was only one dose with the third cycle. We inquired about further episodes of priapism or other sickle cell crises at each subsequent visit, and he did not report any.

## Discussion

We describe a case of a patient with MS on treatment and sickle cell trait who experienced stuttering priapism after prophylactic pegylated filgrastim to prevent prolonged neutropenia after adjuvant chemotherapy for urothelial cancer. To our knowledge, this is the first reported case of priapism triggered by pegylated filgrastim, adding to the limited literature on growth-factor induced vaso-occlusive complications in patients with sickle cell trait. Given the higher rates of malignancies in patients with sickle cell disease compared to the general population, provider and patient awareness of the potential vaso-occlusive risk for this widely used medication is paramount [3].

Sickle cell disease is an inherited hemoglobinopathy of two hemoglobin S genes, whereas sickle cell trait is the presence of one hemoglobin S gene and one normal hemoglobin gene. Sickle cell trait is typically thought to be less symptomatic compared to sickle cell disease. However, more evidence is now supporting that there is increased morbidity in patients with sickle cell trait, including vaso-occlusive crises [1,4,5]. Associations of sickle cell trait with hematuria, renal papillary necrosis, hyposthenuria, splenic infarction, rhabdomyolysis, exercise-related sudden death, venous thromboembolic loss, acute chest, fetal loss, osteonecrosis, and priapism have been reported in the literature, suggesting that even one copy of HbS may have implications for patients [1,4,5].

Priapism is an ischemic event associated with significant pain from compartment syndrome and can be classified as major priapism, lasting more than four hours, or stuttering priapism, lasting less than four hours. Priapism has been reported at least once in 33% of adolescents and adults with sickle cell disease, with the majority being stuttering priapism events, and can be seen with sickle cell trait as well [1,6]. It is associated with increased mental health morbidity, as well as increased sexual dysfunction, and almost half of the patients suffering from priapism related to sickle cell disease do not seek medical attention [6]. Both major and stuttering priapism are caused by stasis of sickled erythrocytes within the sinusoids of the corpora cavernosa [7]. Molecularly, endothelial nitric oxide (NO) is depleted by chronic hemolysis, leading to decreased cyclic guanosine monophosphate (cGMP) and phosphodiesterase type 5 (PDE-5) [7-9]. Depletion of NO, cGMP, and PDE-5 contributes to the inability to terminate the erection, resulting in both major and stuttering priapism [8,9]. Although the mechanism linking pegylated filgrastim to priapism has not been established, in vitro studies have demonstrated inhibition of nitric oxide synthesis with granulocyte-colony stimulating factor in vitro [10]. Other hemoglobinopathies, including beta thalassemia, as well as other hemolytic anemias have also been associated with priapism [11].

Multiple pharmacologic triggers of priapism have been reported in the literature, including antihistamines, anesthetics, antihypertensives, anticonvulsants, alpha-adrenergic blockers, anticoagulants, sex hormones, antipsychotics, antidepressants, vasodilators, medications for attention-deficit disorders, and recreational drugs [12]. To our knowledge, we report the first case of priapism associated with growth factor use. Pegylated filgrastim has been associated with vaso-occlusive crisis in the post-marketing experience, and the prescribing information available from the Federal Drug Administration suggests that severe and sometimes fatal sickle cell crises can occur in patients with sickle cell disorders [13]. The mechanism is poorly understood; current hypotheses suggest that the increase in granulocytes, activation of neutrophils, and cytokine release trigger vaso-occlusion in patients with sickle cell disease [14]. Kasi et al. reported the first case of pegylated filgrastim causing a sickle cell crisis in a patient with sickle cell trait, which manifested as chest pain, shortness of breath, and body aches and was managed successfully as a pain crisis [14]. We add an additional report to this small body of literature with the first case of priapism after pegylated filgrastim in a patient with sickle cell trait.

Management of priapism is multimodal; major priapism is a urologic emergency and immediate involvement of a urologist is indicated for any episode lasting more than three hours [15]. Conservative treatment options include exercise, penile compresses, ice, lying supine, and oral pharmacologic approaches, such as terbutaline, pseudoephedrine, and midodrine, although the efficacy of these treatment options is not well demonstrated [15]. Surgical options include intracavernosal phenylephrine and corporal aspiration. Red blood cell exchange transfusion is not recommended for patients with sickle cell disease as a primary treatment, although it has been reported anecdotally as a preventive strategy [2,15,16].

## Conclusions

Priapism is a manifestation of vaso-occlusive crisis in patients with sickle cell disease and trait. We report the first case, to our knowledge, of priapism presumed to be triggered by pegylated filgrastim in a patient with sickle cell trait and urothelial cancer, although further studies are needed to confirm the association. We aim to increase awareness and promote prompt management in individuals suffering from this disease. Physicians treating patients with sickle cell trait with myelosuppressive chemotherapy should engage in a thorough risk and benefit discussion with patients prior to administration of routine pegylated filgrastim and should inquire about vaso-occlusive crises on each subsequent evaluation of the patient.

## References

[1] Birnbaum BF, Pinzone JJ (2008). Sickle cell trait and priapism: a case report and review of the literature. Cases J.

[2] Ebraheem MS, Verhovsek M (2021). Stuttering priapism in a patient with sickle cell trait treated with automated red cell exchange transfusion. Blood Adv.

[3] Seminog OO, Ogunlaja OI, Yeates D, Goldacre MJ (2016). Risk of individual malignant neoplasms in patients with sickle cell disease: English national record linkage study. J R Soc Med.

[4] Brown TS, Lakra R, Master S, Ramadas P (2023). Sickle cell trait: is it always benign?. J Hematol.

[5] Tsaras G, Owusu-Ansah A, Boateng FO, Amoateng-Adjepong Y (2009). Complications associated with sickle cell trait: a brief narrative review. Am J Med.

[6] Idris IM, Abba A, Galadanci JA (2020). Men with sickle cell disease experience greater sexual dysfunction when compared with men without sickle cell disease. Blood Adv.

[7] Broderick GA, Kadioglu A, Bivalacqua TJ, Ghanem H, Nehra A, Shamloul R (2010). Priapism: pathogenesis, epidemiology, and management. J Sex Med.

[8] Idris IM, Burnett AL, DeBaun MR (2022). Epidemiology and treatment of priapism in sickle cell disease. Hematology Am Soc Hematol Educ Program.

[9] Bivalacqua TJ, Musicki B, Kutlu O, Burnett AL (2012). New insights into the pathophysiology of sickle cell disease-associated priapism. J Sex Med.

[10] Deetjen C, Frede S, Smolny M, Seibel M, Schobersberger W, Hoffmann G (1999). Inhibition of inducible nitric oxide synthase gene expression and nitric oxide synthesis in vascular smooth muscle cells by granulocyte-colony stimulating factor in vitro. Immunopharmacology.

[11] Sardar S, Ali EA, Yassin MA (2021). Thalassemia and priapism: a literature review of a rare association. Cureus.

[12] Idris IM, Abba A, Galadanci JA (2022). Incidence and predictors of priapism events in sickle cell anemia: a diary-based analysis. Blood Adv.

[13] (2024). Administration FaD. Neulasta Prescribing Information. https://www.accessdata.fda.gov/drugsatfda_docs/label/2019/125031s198lbl.pdf.

[14] Kasi PM, Patnaik MM, Peethambaram PP (2013). Safety of pegfilgrastim (neulasta) in patients with sickle cell trait/anemia. Case Rep Hematol.

[15] Bivalacqua TJ, Allen BK, Brock GB (2022). The diagnosis and management of recurrent ischemic priapism, priapism in sickle cell patients, and non-ischemic priapism: an AUA/SMSNA Guideline. J Urol.

[16] Anele UA, Le BV, Resar LM, Burnett AL (2015). How I treat priapism. Blood.

